# Wait, scan and then? Exploring the long-term natural course of 119 low-grade central cartilaginous lesions of the long bones with MRI follow-up

**DOI:** 10.1007/s00330-026-12459-x

**Published:** 2026-03-18

**Authors:** Jacky W. J. de Rooy, Olav Donker, Verena J. J. M. Schrier, H. W. Bart Schreuder, Edwin F. Dierselhuis, Desirée Koopmanschap, Gerjon Hannink, Claudia Deckers, Mathias Prokop, Ingrid C. M. van der Geest

**Affiliations:** 1 Department of Medical Imaging, Radboud University Medical Center, Nijmegen, The Netherlands; 2 Department of Orthopedic Oncology, Radboud University Medical Center, Nijmegen, The Netherlands

**Keywords:** Chondrosarcoma, Enchondroma, Long bones, Atypical cartilage tumor, MRI

## Abstract

**Objectives:**

This study aims to identify MRI characteristics to better understand the natural course of low-grade central cartilage lesions (LG-CCLs) to promote active MRI-based surveillance as an alternative to invasive surgery.

**Materials and methods:**

In this single-center retrospective cohort study, baseline and > 48-month follow-up MRIs of 119 patients with a solitary LG-CCL of the long bones were retrospectively analyzed. None of the included cases had aggressive MRI characteristics, nor had biopsy. Two observers assessed MRI characteristics, length, cortical scalloping, intralesional fat entrapment and fat replacement. LG-CCLs were classified as in regression, stable or progressive. Statistical analysis was assessed using the Kruskal–Wallis rank sum test and Fisher’s exact test. Interobserver agreement was calculated using Cohen’s Kappa coefficient and Intraclass Correlation Coefficient.

**Results:**

The majority of LG-CCLs were labeled as in regression (78/119; 66%) or stable (27/119; 23%). 108/119 patients (91%) showed initial fat entrapment; 56/119 (47%) developed increased fat entrapment and 80/119 (67%) developed fat replacement. Out of 14 patients (median age 30) with tumor growth (14/119; 12%), nine had initial fat entrapment (5/9 also developed fat replacement), and two developed fat entrapment. One LG-CCL with developing fat entrapment and fat replacement showed new scalloping, and two LG-CCLs showed growth and new scalloping without any fat. None developed aggressive MRI characteristics. Interobserver agreement varied from substantial to near perfect agreement, except for moderate agreement on scalloping at baseline.

**Conclusion:**

This study on the natural course of LG-CCLs of the long bones shows that increasing fat entrapment and/or fat replacement over time on MRI may help prognosticate benign biological behaviour and might be supportive for watchful waiting over surgical treatment.

**Key Points:**

***Question*** *Can specific MRI features help identify benign behavior in low-grade central cartilage lesions of long bones to support MRI-based surveillance?*

***Findings*** *Most low-grade lesions were stable or regressed (89%); increasing fat entrapment and/or fat replacement were associated with a benign natural course on MRI*.

***Clinical relevance*** *MRI features like fat entrapment and fat replacement support safe non-operative surveillance of LG-CCLs, avoiding unnecessary surgery*.

**Graphical Abstract:**

**Figure:**
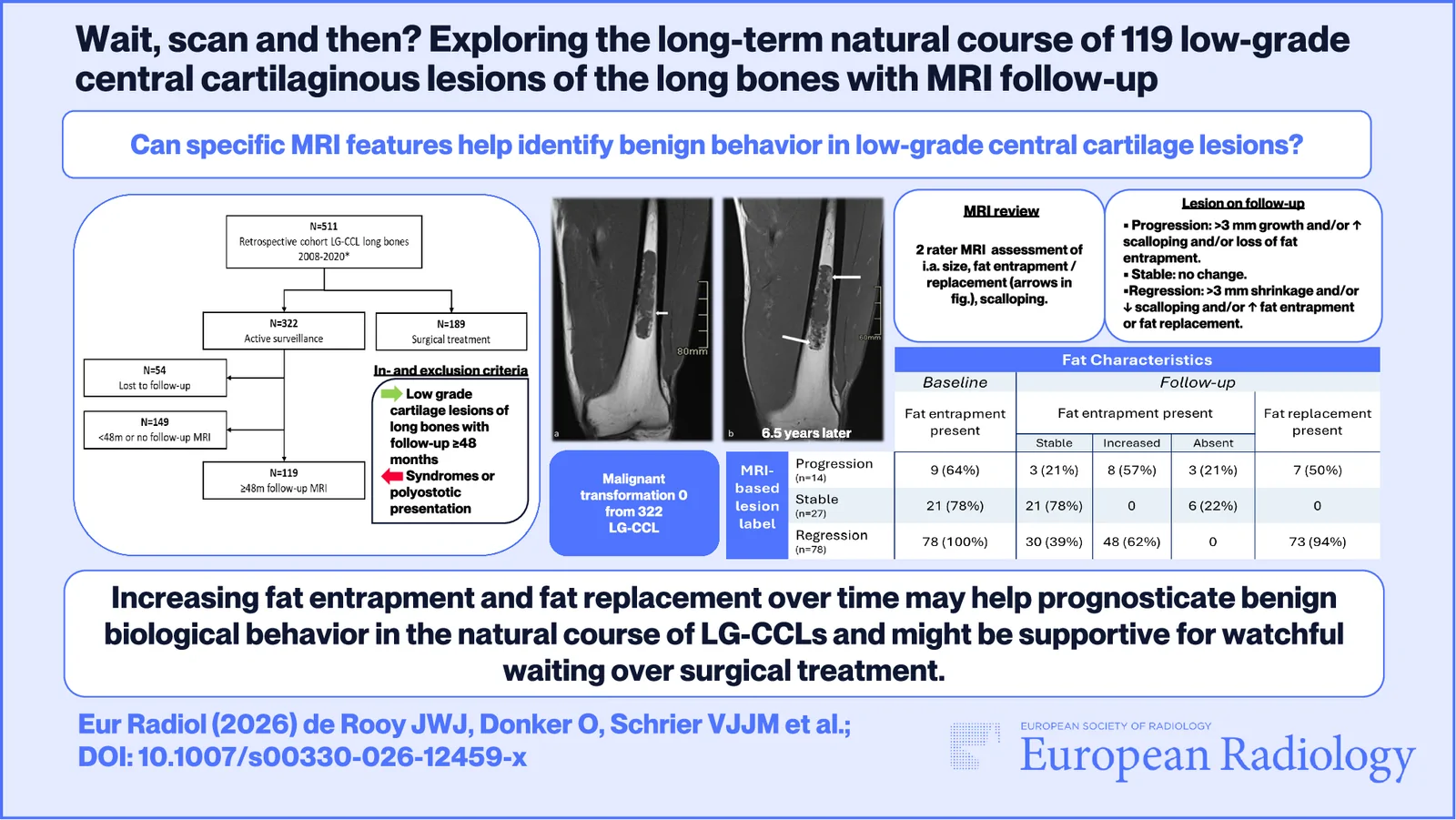

## Introduction

Primary solitary central cartilaginous tumors (CCTs) of the long bones are represented by hyaline cartilage-producing tumors and histopathological grading runs the gamut from benign enchondroma (EC), via intermediate atypical cartilaginous tumor (ACT) to high grades II/III and dedifferentiated chondrosarcoma (CS). High-grade chondrosarcomas (HG-CS) account for 20–25% of primary malignant bone tumors and are the second most common after osteosarcoma [1].

The term ACT was introduced in 2013 by the World Health Organization (WHO) to describe the histologically identical grade I CS more accurately, due to its intermediate character with very low to no propensity to metastasize (0 to < 1%) [2–4]. From 2020, the WHO decided to reserve the term ACT only for CCTs in the appendicular skeleton, leaving grade I CS for those located in the axial skeleton, reflecting the more aggressive behavior of the latter as is commonly seen at axial sites [5].

Radiological as well as histopathological distinction of CCTs, however, can be quite challenging with considerable interobserver variability, especially when it comes to differentiating between benign EC from intermediate ACT [6–9]. Due to the WHO downgrading, the urge to differentiate benign EC and intermediate ACT in the long bones preoperatively gradually shifted toward differentiating both EC and ACTs, which might take advantage of active MRI surveillance, from higher grades of CS, which definitely need *en bloc* resection [10–12].

At the same time, the increasing demand for medical imaging over the past decades, as well as a generally aging population, led to a remarkable increase in incidental detection of EC and ACT in the long bones on routine MRI examinations [13–16]. This is not the case for HG-CS, where patients are more likely to present with clinical symptoms like pain due to bone expansion, cortical destruction and/or thickening with soft tissue extension, perilesional edema and periostal reaction, and bone destruction [17].

The former assumption was that higher grades of CS might develop from ACTs in about 4% of cases [18]. At present, risk estimation of conversion of ACTs of the long bones to malignant higher grades is assumed to be less than 1%, but there are still no known observational studies that report on the risk of progression [13, 14, 19].

The indolent behavior of ACTs has recently led to more studies suggesting wait-and-see approaches, instead of invasive surgical intervention consisting of curettage with local adjuvant therapy or even wide resection [20–23]. Surgery has well-known complications such as wound infection, post-operative hemorrhage, fracture and pain in up to 4% of patients [24]. Most of these studies on watchful waiting proposed different MRI follow-up schemes, and none included long-term results.

A systematic review on MRI characteristics that might help to differentiate EC and ACT from HG-CS concluded that extraosseous expansion with a soft tissue component and cortical breakthrough was more prevalent in HG-CS and that entrapped fat presented more often in ACT, but also mentioned a considerable amount of heterogeneity [25].

This prompted research to focus on MRI features of EC and ACT in the long bones that might help to predict benign biological behavior of these lesions, thus supporting conservative treatment.

For this purpose, several proposals for predictive MRI follow-up classification systems have already been introduced and adjusted, including the Birmingham Atypical Cartilage Tumor Imaging Protocol (BACTIP) and Fibular Atypical Cartilage Tumor Imaging Protocol (FACTIP) [19, 26, 27]. However, adoption in daily clinical practice appears to be challenging.

We investigated ECs and ACTs centrally located in a long tubular bone characterized by a lack of pain symptoms and a lack of MR imaging features suggesting a potential malignancy, which we call a low-grade central cartilaginous lesion (LG-CCL). This study aims to determine MRI characteristics of LG-CCLs that contribute to the understanding of the natural course of LG-CCLs of the long bones during long-term active MRI surveillance and could support watchful waiting over surgical treatment.

## Materials and methods

### Study design and patient selection

This single-center, retrospective observational cohort study was approved by the institutional review board, which granted a waiver for written informed consent.

An observational cohort of patients with a baseline MRI diagnosis of LG-CCL of the long bones between 2008 (introduction of digital health records in our hospital) and May 2020 was retrospectively analyzed. Follow-up MRI was included up to May 2024.

Radboud UMC is a tertiary referral center in the Netherlands for bone- and soft tissue tumors. During the first visit in our academic center, patients are informed about the diagnosis of an LG-CCL, about the latest scientific findings and about the suggested treatment options for their specific lesion. The definitive choice of treatment is always made in collaboration with the patient as part of Shared Decision Making.

Inclusion criteria for this study were as follows: MRI diagnosis of an LG-CCL in a long tubular bone, with no malignant imaging characteristics (i.e., bone expansion, cortical destruction/thickening, soft-tissue mass, perilesional edema, osteolysis or aggressive periosteal reaction) at baseline and no pain in the region of the tumor. Patients had to have at least 2 MRIs with a minimum of 48 months between baseline diagnosis and last follow-up. Patients were excluded in case of Maffucci syndrome or Ollier’s disease or polyostotic presentation. To prevent potential interference with the interpretation of lesion characteristics, patients were also excluded if a biopsy or previous surgery had been performed. Patients underwent active surveillance when no malignant imaging characteristics (i.e., cortical destruction, soft-tissue mass, osteolysis or aggressive periosteal reaction) were present at baseline and if there was no pain related to the tumor.

Patient-specific follow-up schedules had been designed in line with the Dutch national consensus. Over the last couple of years, this consensus has changed to a more conservative and wait-and-see approach. If a lesion proved to be stable after several years of follow-up, patients were discharged from further follow-up and were instructed to reach out in case of increasing symptoms, i.e., localized pain and/or tumor-related pain. A query on histopathological data in the Dutch national pathology database (PALGA) was performed in all cases diagnosed with LG-CCL in the inclusion period, also including those lost to follow-up, to identify patients in whom HG-CS had been diagnosed since baseline.

### Data acquisition and MR imaging analysis

Patient-related data, including age at time of diagnosis, gender and maximum follow-up time, were extracted from the electronic health record using CTcue software and manually added into a pseudonymized electronic data collection system (EDC Castor).

MRI screening in our hospital was performed on a 1.5-T MRI scanner (Siemens Healthineers, Symphony (Tim)/Avanto (Fit)). The assessment of the LG-CCL was made on 3–5 mm contiguous axial sections, 3–4 mm contiguous coronal or sagittal T1-weighted TSE sections and axial 3–5 mm contiguous T2-weighted TSE sections with fat-saturation. All MRIs were scored by a senior resident, specializing in MSK radiology with four 4 of experience (V.F.S., OBS1) and an experienced musculoskeletal radiologist (J.d.R., OBS2) with more than 25 years of experience in radiological assessment in orthopedic oncology. In case of discrepancies, a consensus method was applied.

Tumor characteristics that were scored included: location of the lesion, maximum craniocaudal size, presence of endosteal scalloping, presence of fat entrapment and presence of fat replacement. All scores were evaluated with a discussion-based consensus opinion.

**Fig. 1:**
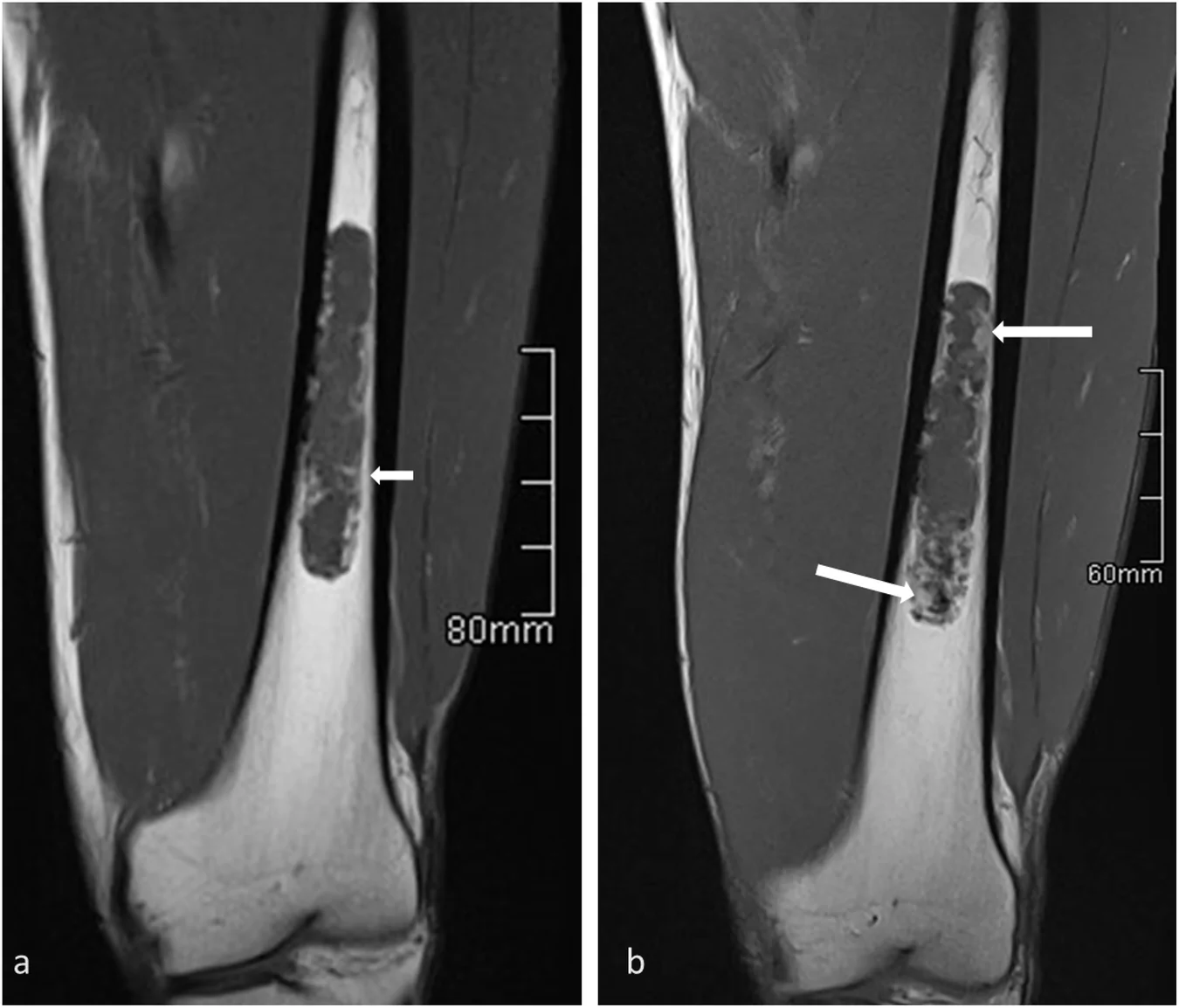
**a** TSE T1-weighted coronal baseline MRI showing small spots of fat entrapment (small arrow) in an LG-CCL and (**b**) follow-up MRI 6.5 years later; arrows pointing at fat replacement. LG-CCL, Low-grade central cartilaginous lesion

Craniocaudal size was measured on coronal or sagittal T1-weighted images. Endosteal scalloping was defined as loss of inner cortical bone and was assessed according to the BACTIP protocol on the axial T1-weighted slice with the greatest lesion involvement as no scalloping, < 10% scalloping of the circumferential cortex (less than 36°) and ≥ 10% scalloping of the circumferential cortex [19]. Fat entrapment was assessed simultaneously on axial and coronal or sagittal T1-weighted images as the presence of high signal intensity foci correlating with fat within the otherwise T1-intermediate signal of cartilage. Fat replacement was defined as the replacement of cartilage lobules by fatty marrow on follow-up MRI (Fig. 1).

Lesions on the latest follow-up MRI were compared with lesions on baseline MRI and were labeled as either ‘stable,’ ‘progression’ or ‘regression.’ Progression was defined as: > 3 mm of craniocaudal growth and/or an increase in scalloping and/or the loss of fat-entrapment. Regression was defined as: > 3 mm of craniocaudal shrinkage and/or decreased scalloping and/or an increase in fat-entrapment and/or fat replacement of the lesion. A cut-off value of 3 mm was set as a compromise between sensitivity for change and measurement variability. In lesions labeled as ‘progression,’ all available MRIs were evaluated to be able to assess the change in growth rates and MR characteristics.

### BACTIP/FACTIP classification

In addition to our criteria, BACTIP/FACTIP classification was applied to all lesions located around the knee (including also distal femur metadiaphysis and proximal tibia metadiaphysis) or the proximal humerus and evaluated over time using their definition of MRI change in longitudinal length > 1 cm [26, 27]. Lesions located in the proximal femur, distal humerus and distal tibia (17/119, 14.3%) were therefore excluded from this classification.

### Statistical analysis

Statistical analysis of our data was performed with R (version 4.4.2; R Foundation for Statistical Computing). All individual patients were labeled as ‘progression,’ ‘stable,’ or ‘regression,’ based on our criteria. Baseline characteristics and other collected variables were compared between groups using Kruskal–Wallis test for continuous variables and Chi-Squared test for categorical variables. A *p*-value of < 0.05 was considered statistically significant.

Interobserver agreement on fat entrapment, fat replacement and scalloping was calculated using Cohen’s Kappa coefficient. Interobserver agreement on tumor length measurement was calculated using the intraclass correlation coefficient (ICC). For both, 95% confidence intervals (CIs) were reported. Interpretation of kappa values followed the Landis and Koch criteria, with values < 0.20 indicating slight, 0.21–0.40 fair, 0.41–0.60 moderate, 0.61–0.80 substantial, and > 0.80 almost perfect agreement [28]. ICC values were interpreted according to the guidelines proposed by Koo and Li, with values < 0.5 considered poor, 0.5–0.75 moderate, 0.75–0.9 good, and > 0.9 excellent reliability [29].

## Results

### Patient demographics

**Fig. 2:**
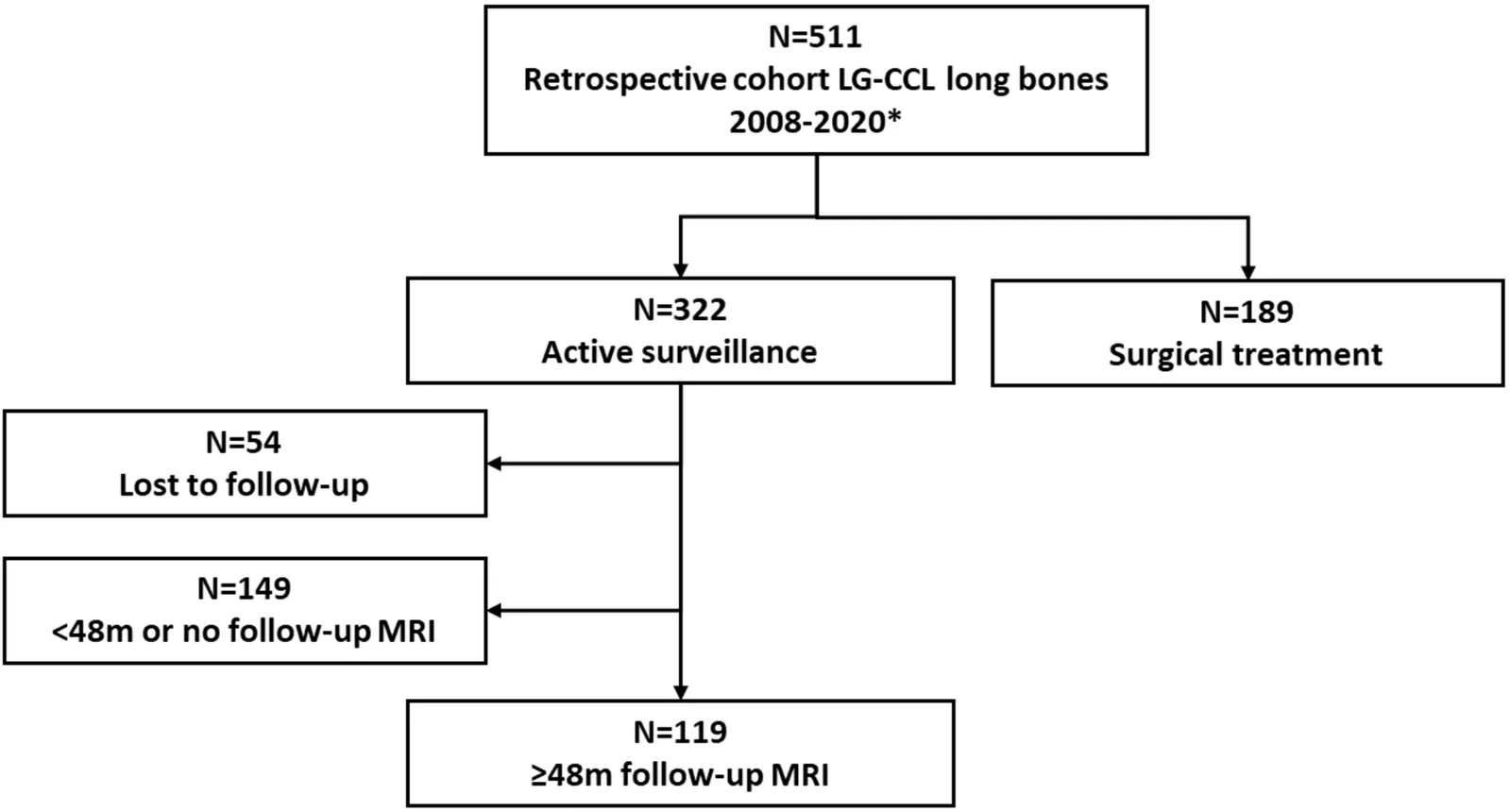
Flowchart of included patients and those lost to follow-up. * Data inclusion concluded on April 30th 2020. LG-CCL, Low-grade central cartilaginous lesion

**Fig. 3:**
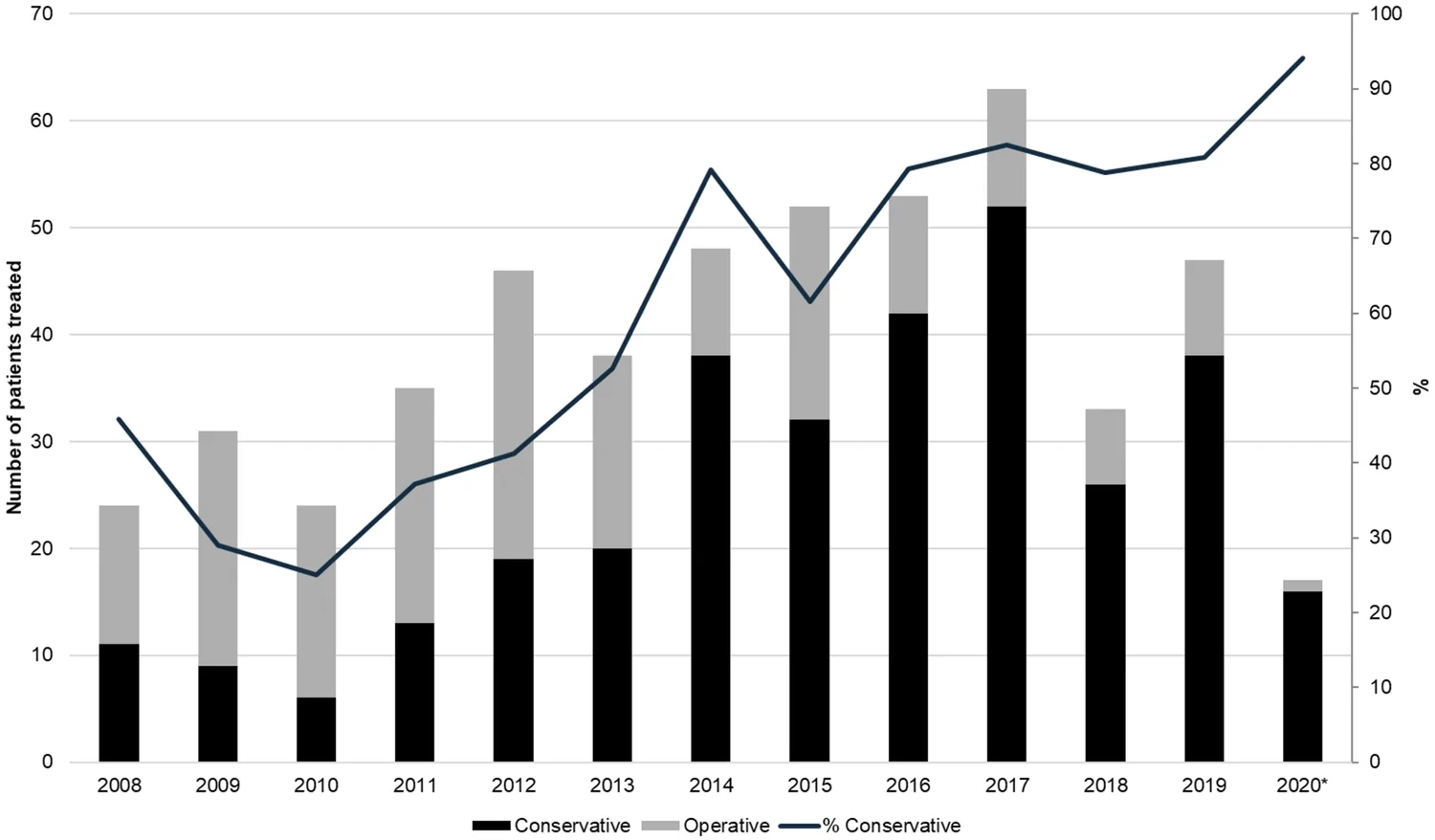
Total number of low-grade central cartilaginous tumors located in the long bones treated at our tertiary referral center. * 2020 only represents months between January and April

In total, 322 out of a cohort of 511 patients with a low-grade central cartilaginous tumor in a long bone (63%) were treated conservatively between January 1st, 2008, and April 30th, 2020. At the time of writing, 54 out of 322 patients (17%) were lost to follow-up. None of the 322 patients, including those lost to follow-up, presented with a histopathological diagnosis of HG-CS in the Dutch national pathology database. Among those 322 patients, 119 (37%) met the inclusion criteria for further analysis in this study. A tree diagram showing participant selection for this study is presented in Fig. 2. An overview of treatment in our tertiary referral center in recent years is shown in Fig. 3 and shows a clear trend toward a more conservative approach over time.

Patient and imaging characteristics across the groups with progression, stable disease and regression are presented in Table 1. Mean follow-up time across the cohort was 72 months (range 48–160). Mean age of the included 119 patients (80 female; 67.2%) was 51 years at diagnosis (range 18–82 years). Median age in the ‘progression’group was 30 years [IQR 22–40] and significantly lower (*p* < 0.001) than both the age in the ‘stable’ group with 54 years [IQR 46–63] and the ‘regression’ group with 56 years [IQR 46–62]. The mean time of follow-up across the cohort was 72 months (range 48–160).

**Table 1:** Patient demographics and MR imaging characteristics of low-grade central cartilaginous lesions and their development during FU in the progression, stable and regression group

		Progression	Stable	Regression	*p*-value^a^
*N* (% of total)		14 (11.8%)	27 (22.7%)	78 (65.5%)	
Follow-up (months), median (IQR)		74 (60–115)	58 (49–67)	67 (56–82)	0.01
Age (years), median (IQR)		30 (22–40)	54 (46–63)	56 (46–62)	< 0.001
Female (%)		8 (57.1%)	20 (74.1%)	50 (64.1%)	0.50
Size MRI baseline (mm), median (Q1, Q3)		30 (20, 50)	33 (21, 57)	45 (36, 68)	0.02
Size MRI last follow-up (mm), median (Q1, Q3)		42 (34, 68)	34 (21, 59)	45 (34, 64)	0.83
Growth (mm), median (Q1, Q3)		8.50 (7, 17)	0 (0, 1)	0 (−3, 0)	< 0.001
Scalloping baseline (%)					0.28
	No	10 (71.4%)	12 (44.4%)	39 (50%)	
	< 10%	2 (14.3%)	10 (37.0%)	31 (39.7%)	
	> 10%	2 (14.3%)	5 (18.5%)	8 (10.3%)	
Scalloping last follow-up (%)					0.008
	Stable	4 (28.6%)	15 (55.6%)	39 (50%)	
	Increased	3 (21.4%)	0	0	
	Absent	7 (50%)	12 (44.4%)	39 (50%)	
Fat entrapment = yes (%)		9 (64.3%)	21 (77.8%)	78 (100%)	< 0.001
Fat entrapment last FU (%)					< 0.001
	Stable	3 (21.4%)	21 (77.8%)	30 (38.5%)	
	Increased	8 (57.1%)	0	48 (61.5%)	
	Absent	3 (21.4%)	6 (22.2%)	0	
Fat replacement = yes (%)		7 (50%)	0 (0%)	73 (93.6%)	< 0.001
Location, *n* (%)					0.08
	Humeral metadiaphysis (proximal)	0	0	4 (5.1%)	
	Proximal humerus	2 (14.3%)	6 (22.2%)	30 (38.5%)	
	Proximal femur	0	4 (14.8%)	12 (15.4%)	
	Femoral metadiaphysis (distal)	2 (14.3%)	3 (11.1%)	7 (9.0%)	
	Distal femur	8 (57.1%)	9 (33.3%)	18 (23.1%)	
	Proximal tibia	1 (7.1%)	2 (7.4%)	2 (2.6%)	
	Distal tibia	1 (7.1%)	0	0	
	Proximal fibula	0	3 (11.1%)	5 (6.4%)	
BACTIP/FACTIP, *n* (%)					0.09
	IA	6 (42.9%)	5 (18.5%)	9 (11.5%)	
	IB	1 (7.1%)	1 (3.7%)	8 (10.3%)	
	IC	0	4 (14.8%)	2 (2.6%)	
	IIA	3 (21.4%)	5 (18.5%)	22 (28.2%)	
	IIB	1 (7.1%)	6 (22.2%)	20 (25.6%)	
	IIC	2 (14.3%)	2 (7.4%)	5 (6.4%)	

^a^ Kruskal–Wallis rank sum test; Fisher’s exact test for count data with simulated *p*-value (based on 2000 replicates); Pearson’s Chi-squared test

*BACTIP* Birmingham atypical cartilage tumor imaging protocol, *FACTIP* Fibular atypical cartilage tumor imaging protocol, *FU* follow up

### MR imaging analysis

MR imaging analysis data are included in Table 1. A total of 78 lesions (65.5%) were labeled as regression, 27 (22.7%) as stable and only 14 lesions (11.8%) were labeled as progression. Median follow-up in the S group was significantly shorter than in the other two groups.

The lesion sizes (median [Q1, Q3]) in the Stable (33 mm, [21 mm, 57 mm]) and the Progression (30 mm [20 mm, 50 mm]) group were significantly smaller compared to the Regression group (45 mm [36 mm, 68 mm]) at baseline MRI. There was no significant difference in scalloping between the three groups, neither at baseline nor on the last follow-up MRI. A decrease in scalloping was not found in any lesion in our entire cohort.

There was a significant increase in fat entrapment (*p* < 0.001) in both the Regression group (64%) as well as the Progression group (57%) on follow-up MRI. Loss of fat entrapment was not found in any lesion in our entire cohort. The replacement of cartilage lobules by fatty marrow (fat replacement) on follow-up MRI was also significantly more often present (*p* < 0.001) in the Regression group compared to the Progression group, 93.6% vs 50%.

The interobserver agreement is shown in the supplementary material (Table S1). Cohen’s Kappa varied from the upper level of substantial agreement (fat entrapment at baseline, κ = 0.80 (95% CI 0.61–0.99)) to near perfect agreement (fat entrapment at follow-up, κ = 0.82 (95% CI 0.73–0.92); fat replacement, κ = 0.84 (95% CI 0.74–0.94)), except for moderate agreement on scalloping at baseline (κ = 0.45 (95% CI 0.31–0.59)). The agreement on scalloping at follow-up did not improve, but the only three cases with new or progression of scalloping were detected by both observers. ICC for size on baseline MRI and follow-up MRI was near perfect.

**Fig. 4:**
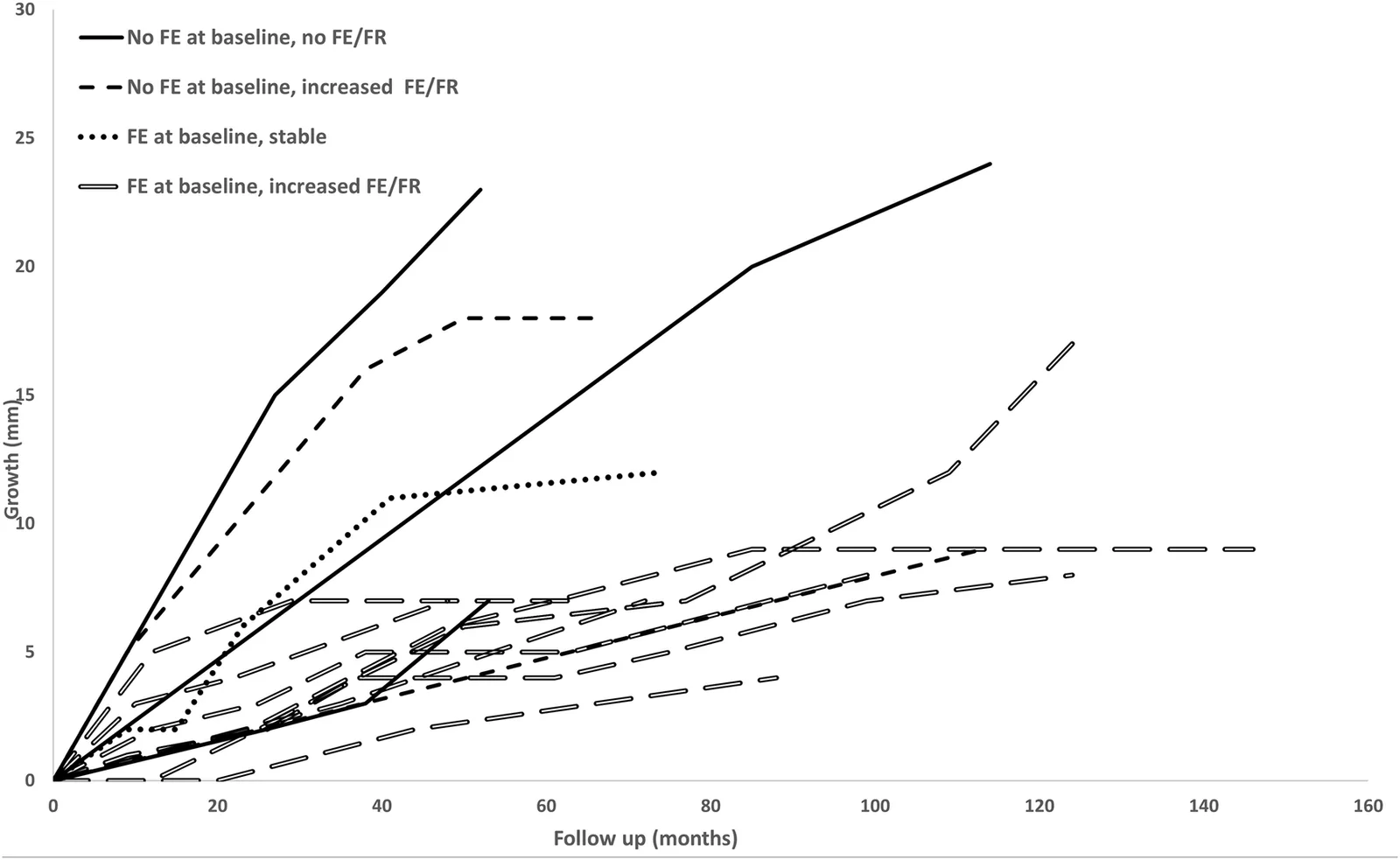
Spaghetti plot of growth in time of all lesions in the Progression group. FE, fat entrapment; FR, fat replacement

None of the 14 patients in the Progression group showed signs of malignant transformation to a HG-CS. Changes in size, fat entrapment and fat replacement are graphically shown in Fig. 4. Only two patients showed a steady growth rate over time. These patients were among the youngest in our cohort (20 and 22 years) and showed no fat entrapment or fat replacement in any of their MRI scans. All other patients with progression showed a decrease in growth rate from the first two to the final two scans.

### BACTIP and FACTIP

A total of 102 patients (86%) could be classified according to BACTIP or FACTIP at baseline (Table 1). Five of fourteen patients in our Progression group would have been classified as progressive if the BACTIP/FACTIP parameter of > 1 cm was used in addition to our threshold > 3 mm.

**Fig. 5:**
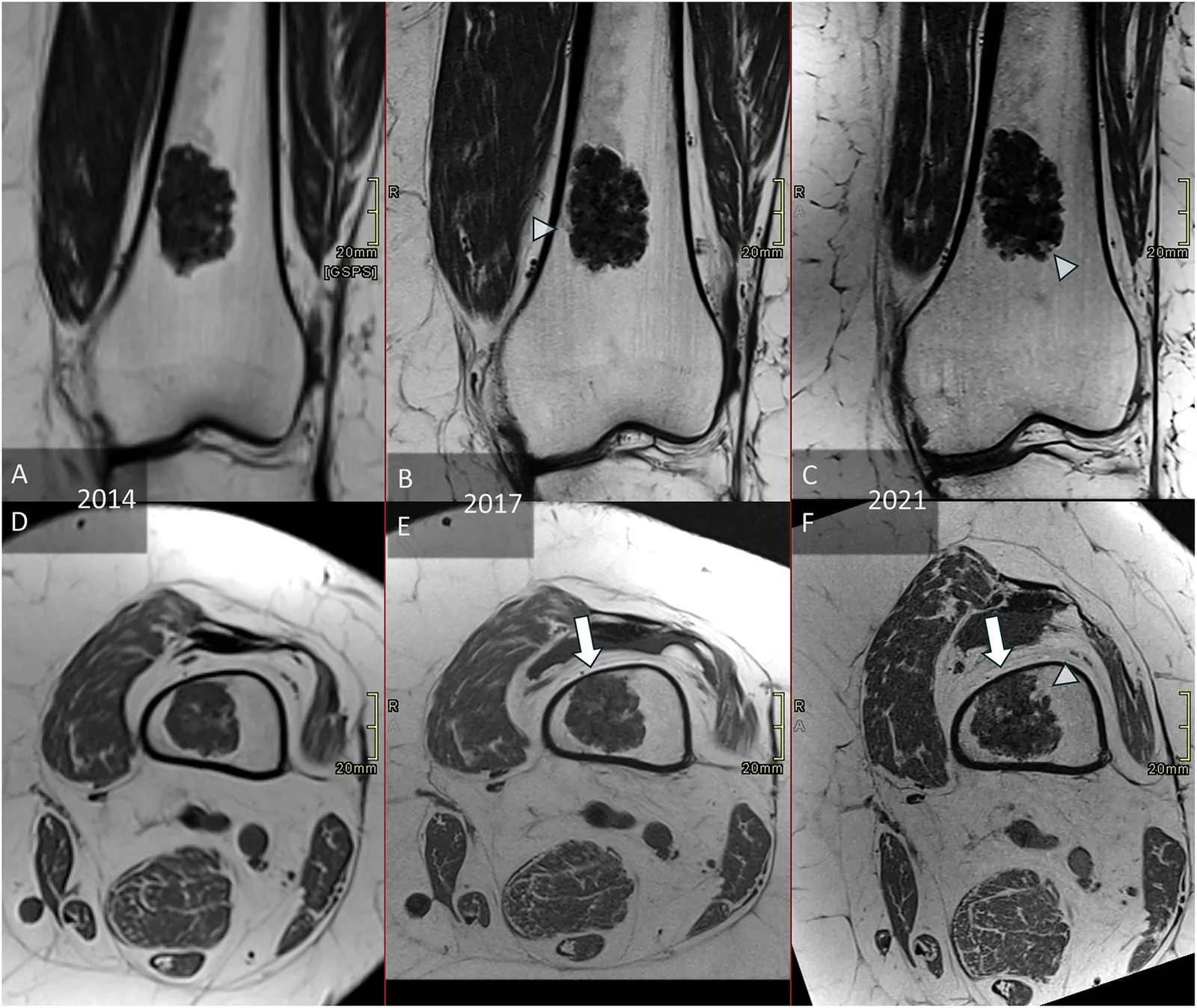
TSE T1-weighted coronal and axial images of LG-CCL (**A**, **D**) with new scalloping (**E**, **F** arrow) and signs of increasing fat entrapment over time and fat replacement (**B**, **C** and **F**, arrowhead). Cranio-caudal length has been measured on different slices focusing on maximum lesion size and is therefore not shown here. LG-CCL, Low-grade central cartilaginous lesion

Three of 14 LG-CCLs in our Progression group would have received no follow-up (3%) BACTIP. Two of these LG-CCLs were found in the distal femur of females aged 22 and 20 and were classified as BACTIP category IA because of > 1 cm growth. These two lesions were those that showed a steady growth rate over time (Fig. 4). The third case developed new scalloping after 6 years. It involved a BACTIP category IIA LG-CCL in the distal femur in a female patient aged 56 years. The lesion revealed fat entrapment at baseline, with increasing fat entrapment after 7 years and the appearance of fat replacement over 3–7 years (Fig. 5). The craniocaudal length of this lesion increased by 8 mm over time. All three cases developed focal endosteal scalloping over time, the only three of the entire patient group.

### Surgery

Of the 119 patients who had at least 48 months of watchful waiting, 3 patients (2.5%) eventually received surgery after this period of wait-and-see, as is shown in Table 2. The reason for surgery was a subtle growth in 2 cases and the placement of a knee prosthesis in 1 case. Histopathology showed EC or ACT in all cases. There were no perioperative complications in any of these patients.

**Table 2:** Surgical cases

Age at diagnosis	Location	Initial size	Growth (CC in mm)	Reason for surgery	Time until surgery (months)	Pathology
38	Tibia (distal)	32	6	Growth	53	ACT
69	Femur (distal)	32	1	Prosthesis	57	Enchondroma
22	Femur (distal)	34	12	Growth	55	Enchondroma

*ACT* Atypical cartilaginous tumor

## Discussion

This study investigated the natural course of LG-CCLs of the long bones and MRI characteristics that contribute to the understanding of their biological behavior. Approximately 66% of the LG-CCLs showed MR signs of regression over time. Among the features that indicate regression, only increasing fat entrapment and/or secondary fat replacement at follow-up were present in all these lesions. None of the lesions showed decreased scalloping. All lesions had displayed fat entrapment at baseline, which might therefore be a reliable parameter in the prediction of indolent benign biological behavior of LG-CLLs, further confirming and strengthening the results of the mid-term study by Deckers et al [21]. The definition of regression in our study, with focus on fat entrapment and fat replacement, next to tumor length and scalloping, might be an explanation for the unusual high prevalence of regression, compared to Woltsche et al, reporting regression varying between 11.8% (around the knee) and 13.8% (shoulder), focusing on tumor size only as a measure of regression of 182 cartilaginous lesions within a much shorter median follow-up period [30]. The only other previous study focusing on fat replacement and tumor size by Chung et al also found a higher prevalence of regression of 52.4%, albeit in a much smaller sample size [31]. Nearly 23% remained stable, and 12% showed some progression, although none of the LG-LCCs developed any signs of malignant progression, i.e., bone expansion, cortical destruction, soft tissue mass and/or aggressive periosteal reaction.

There is only sparse literature on the biological behavior of LG-CCLs of the long bones using MRI follow-up with emphasis on intralesional fat. A single case report, a small short-term study on 21 patients by Chung et al and our midterm study with a minimum follow-up of 24 months showed evidence for regression of LG-CCLs over time with progression of fat entrapment as well as replacement of cartilaginous lobules by fat [21, 31, 32]. An earlier study by Vanel et al already stated that fat entrapment in cartilaginous lesions can be reliably detected on MRI and can be useful in the differentiation of benign from malignant tumors [33]. Other studies reported that entrapped fat was significantly more observed in ACTs than HG-CS [34, 35]. Loss of fat entrapment on MRI, on the contrary, has been mentioned as a reliable sign of malignancy by both known systematic reviews on MRI characteristics that might help in the differentiation between HG-CS and ACT [25, 36].

The maximum cranio-caudal length and the absence or presence of scalloping on MRI in accordance with BACTIP and FACTIP recommendations were evaluated, as we also aimed to explore the contribution of those parameters to the natural course of LG-CCLs in our group [19, 26]. The minimum long-term MRI follow-up time span of 48 months (4 years) also fits into these protocols.

No clinically relevant size difference was noticed between the three groups. The cut-off value of 4 cm in BACTIP/FACTIP has been chosen pragmatically by the authors as a risk-averse safe number, whereas 5 cm is often used in literature as a cut-off value between EC and LG-CS [17, 19]. These values have all been set before the downgrading of ACT by the WHO. Measurements of scalloping in our study reflect results of previous studies, reporting a lack of correlation between endosteal scalloping and biological aggressiveness [20, 37].

Measurement of scalloping at baseline, nevertheless, is not useless, as the development of scalloping or increasing scalloping over time indicates progression of an LG-CCL. The moderate interobserver agreement on scalloping in our study might reflect difficulty in delineating lesion boundaries from cortical bone on MRI. CT can be a useful complementary tool to assess scalloping, but according to Crim et al, scalloping on its own should not be considered an independent sign of chondrosarcoma but is rather correlated to large size and/or subcortical location [8].

In our study, the cut-off value of > 3 mm for growth, i.e., progression, was chosen, considering possible measurement errors, because the key objective of this study was to evaluate the natural course. Measurement errors might be caused by differences in slice thickness and subtle variations in obliquity on coronal or sagittal images on serial MRIs. There is, however, no consensus on growth in the literature. Sampath Kumar et al defined a cartilaginous lesion with a total increase of > 6 mm in 3 years as active, and a recent study by Woltsche et al reported different growth rates for ECs and ACTs, interpreting an increase of 1 mm as growth, independent of follow-up time [20, 30]. According to the BACTIP protocol, Patel et al defined growth as an increase of > 1 cm over 1 year to a maximum of 4 years [19]. Given the BACTIP parameter of growth > 1 cm, only 5 out of 14 patients would remain in our progression group. BACTIP has been evaluated recently retrospectively and externally by Van Den Berghe et al in a very heterogeneous group of 123 patients, including 49 LG-CCLs and 74 HG-CCLs [38]. They proposed minor changes to cut-off values when using BACTIP, in order to avoid diagnostic delay in five ACTs and one HG-CCL (roughly 5% of their patients). Results of our study reveal that we should be cautious to discharge young patients with small LG-CCLs without fat entrapment from any follow-up. Of course, there were many LG-CCLs in our study with an unnecessarily long period of follow-up, as the emphasis was on the natural behavior of those lesions.

Despite the slowly increasing number of publications on conservative treatment with active MRI surveillance of LG-CCLs over the last 5 years, there is, however, still no global consensus on the follow-up and management of these lesions [11, 14, 20, 21, 23, 39]. At the recent 2024 Birmingham Orthopedic Oncology Meeting, 300 participants from over 50 countries attempted to gain global consensus on cartilaginous lesions, but especially the management of ACTs remained one of the most controversial areas [29]. This may have been partly driven by the known large interobserver variability in both radiological distinction and histopathological grading of central cartilage lesions, but not in the least, this disagreement might also be caused by country-bounded laws and fear for prosecutions in case of missed HG-CS, albeit the estimated chance of malignant transformation of LG-CCLs around the knee and humerus is still less than 1% [6–8, 14, 40]. Patient preference might play a large role in the treatment of choice, even though this might not always be in compliance with the latest findings [41].

But which indolent LG-CCL of the long bones will show aggressive malignant transformation over probably many, many years? Is it still feasible then to expose patients with an LG-CCL to a lifelong imaging regime or even operative treatment? A question that has also been posed by several other studies [14, 42, 43]. We postulate that additional assessment of the lack of intralesional fat entrapment within an LG-CCL without scalloping will lower false-negative classified lesions in BACTIP/FACTIP.

On the contrary, regressive lesions with increasing fat entrapment and/or fat replacement over time (i.e., > 48 months), whether with or without scalloping < 10% or ≥ 10% at baseline MRI will probably provide clinicians with an extra argument to discharge patients without clinical signs of local pain related to the LG-CCL from follow-up and we recommend to stop watchful waiting. Patients should be instructed to revisit their orthopedic surgeon only in case of new local pain that might be related to renewed activity of the LG-CCL, because it is acknowledged that LG-CCL may evolve in a heterogeneous and sometimes ambivalent manner, with areas of regression coexisting with regions of potential progression [5].

### Limitations

Because this retrospective cohort study was performed at one tertiary referral center, institutional selection bias may have occurred. However, the vast majority of bony lesions are referred to tertiary centers in the Netherlands, reducing institutional bias to a minimum.

The retrospective nature of the study and the lack of a uniform MRI protocol resulted in measurements in longitudinal planes performed on coronal or sagittal slices with thicknesses up to 4 mm. Consequently, our size measurements, and therefore our conclusions regarding size changes, are prone to measurement error due to variable and relatively coarse slice thicknesses compared with current standards. As thin-slice multiplanar reconstructions were not available most of the time, variations in obliquity on coronal or sagittal images on serial MRIs may also have introduced measurement inaccuracies. Additionally, size changes were only analyzed in the longitudinal direction, which may have limited the assessment of growth or progression.

None of the 119 LG-CCLs showed malignant transformation, which confirms that the risk for malignant transformation of such lesions is extremely low, even with an extended surveillance period of a mean of nearly 6 years. However, this does not allow to confirm any risk factors for malignant transformation. Conclusions can only be made heuristically based on the pattern of involution that we found consistently in this group.

We focused on patients with LG-CCLs without aggressive imaging features with a minimum MRI follow-up of 4 years and excluded all operated LG-CCLs, whose biological behavior could not be investigated. The study design and focus on non-aggressive LG-CCLs may account for the unusually high regression rate in our patients. Nonetheless, insufficient knowledge exists on the long-term biology of LG-CCLs to ascribe this entirely to selection bias, even with our follow-up period. Over time, this bias clearly dropped due to the growing trend toward conservative treatment, but we cannot rule out that some excluded lesions with fat entrapment might have progressed to a higher grade. None of the LG-CCLs on active surveillance (*n* = 322) in our study showed malignant transformation to HG-CS, confirmed by additional search in PALGA, the Dutch national pathology database. The study by van Praag et al on a large multicenter cohort of 2168 patients found that increased operative treatment due to increasing incidence of ACTs and EC was not followed by a significant increase in incidence of HG-CCLs, so the exclusion of treated LG-CCLs in our study will likely not impact our findings [13]. Expanding this study to a multicenter design might strengthen the generalizability of results.

To account for the potential loss of data on malignant transformation in the lost to follow-up group, we performed an additional search in PALGA, the Dutch national pathology database, which includes confirmed histopathological diagnoses from other hospitals. Since none of the lost patients have been diagnosed with HG-CS, we hypothesize that no relevant data were missed in this group.

The analyses in this study were restricted to univariable comparisons, focusing on describing differences between outcome groups rather than predicting progression. In our study, the primary analysis aimed to compare baseline characteristics (age, lesion size, location, scalloping, baseline fat) between lesions classified as stable (S), progression (P), or regression (R) on follow-up MRI. Each lesion was individually compared between baseline and follow-up to assign its category. Multivariable modeling could provide additional insights into independent predictors of progression or regression. However, given the relatively small sample size and the limited number of events in each outcome category, multivariable analysis would have limited statistical power and could be prone to overfitting.

## Conclusion

Increasing fat entrapment and fat replacement over time may help prognosticate benign biological behavior in the natural course of LG-CCLs and might be supportive for watchful waiting over surgical treatment. There should still be caution in defining absolute MR parameters of benign behavior until we definitely know which LG-CCLs will evolve to higher-grade CS. Multicenter studies with larger cohorts and longer follow-up time, also focusing on the rare cases of malignant transformation, will help elucidate this. Small lesions without intra-lesional fat entrapment at baseline, especially in patients younger than 40 years, as well as lack of development of fat entrapment and/or fat replacement over time, are biological factors that cannot be ignored and should at least also be included in MRI follow-up schemes to improve diagnostic accuracy.

## Supplementary information

ELECTRONIC SUPPLEMENTARY MATERIAL

## Abbreviations

ACT: Atypical cartilaginous tumor
BACTIP: Birmingham atypical cartilage tumor imaging protocol
CCTs: Central cartilaginous tumors
Cis: Confidence intervals
CS: Chondrosarcoma
EC: Enchondroma
FACTIP: Fibular atypical cartilage tumor imaging protocol
HG-CS: High-grade chondrosarcomas
ICC: Intraclass correlation coefficient
LG-CCL: Low-grade central cartilaginous lesion
