# Supplementary material for: Wait, scan and then? Exploring the long-term natural course of 119 low-grade central cartilaginous lesions of the long bones with MRI follow-up
Source: Eur Radiol. 2026 Mar 18;36(8):6300–11. doi: 10.1007/s00330-026-12459-x (PMC13341817; doi:10.1007/s00330-026-12459-x)
Supplement: Supplementary file 1 — ELECTRONIC SUPPLEMENTARY MATERIAL [file 330_2026_12459_MOESM1_ESM.pdf]

Wait, Scan and Then? Exploring the Long-Term Natural Course of 119 Low-Grade Central Cartilaginous Lesions of the Long Bones with MRI Follow-up

ELECTRONIC SUPPLEMENTARY MATERIAL

Table S1. Cohen's Kappa and Intraclass Correlation Coefficient (ICC) values for interobserver agreement

	**Coefficient (95%CI)**
**Cohen's Kappa**	
Scalloping baseline	0.45 (0.31 - 0.59)
Fat entrapment baseline	0.80 (0.61 - 0.99)
Fat entrapment follow-up	0.82 (0.73 - 0.92)
Fat replacement	0.84 (0.74 - 0.94)
Progression (progression, stable, regression)	0.84 (0.74 - 0.94)
**ICC**	
Tumor length baseline	0.99 (0.99 - 1.00)
Tumor length follow-up	0.99 (0.98 - 0.99)
Tumor length difference	0.79 (0.71 - 0.85)
